# Community-based sero-prevalence of chikungunya and yellow fever in the South Omo Valley of Southern Ethiopia

**DOI:** 10.1371/journal.pntd.0008549

**Published:** 2020-09-03

**Authors:** Adugna Endale, Daniela Michlmayr, Woldaregay Erku Abegaz, Getahun Asebe, James W. Larrick, Girmay Medhin, Mengistu Legesse

**Affiliations:** 1 Aklilu Lemma Institute of Pathobiology, Addis Ababa University, Addis Ababa, Ethiopia; 2 School of Medicine, College of Medicine and Health Sciences, Dire Dawa University, Dire Dawa, Ethiopia; 3 Division of Infectious Diseases and Vaccinology, School of Public Health, University of California Berkeley, Berkeley, California, United States of America; 4 Department of Microbiology, Immunology & Parasitology, School of Medicine, College of Health Sciences, Addis Ababa University, Addis Ababa, Ethiopia; 5 Department of Veterinary Microbiology, Immunology and Public Health, College of Veterinary Medicine, Addis Ababa University, Bishoftu, Ethiopia; 6 College of Agriculture and Natural Resources, Gambella University, Gambella, Ethiopia; 7 Panorama Research Institute, Sunnyvale, California, United States of America; University of Washington, UNITED STATES

## Abstract

**Background:**

Chikungunya (CHIK) and yellow fever (YF) are becoming major public health threats in East African countries including Ethiopia. In Ethiopia, there is no reliable information about the epidemiology of CHIK. This study aimed to assess a community-based sero-prevalence of CHIK and YF in the South Omo Valley, an endemic area for YF.

**Methods:**

Between February and June 2018, blood samples were collected from study participants and screened for IgG antibody against CHIK virus (CHIKV) and YF virus (YFV) infections using ELISA. Data were computerized using Epi Data Software v.3.1 and analyzed using SPSS.

**Results:**

A total of 360 participants (51.7% males, age range from 6 to 80, mean age ± SD = 31.95 ± 14.05 years) participated in this study. The overall sero-prevalence of IgG antibody was 43.6% (157/360) against CHIKV, while it was 49.5% (155/313) against YFV. Out of 155 samples which were positive for IgG antibody to YFV, 93 (60.0%) were positive for IgG antibody to CHIKV. Out of 158 samples which were negative for IgG antibody to YFV, 64(40.5%) were positive for IgG antibody to CHIKV. There was a significant positive correlation between IgG antibodies to CHIKV and YFV (sr = 0.82; P<0.01). Residency in the Debub Ari district (AOR = 8.47; 95% CI: 1.50, 47.74) and travel history to sylvatic areas (AOR = 2.21; 95% CI: 1.02, 4.81) were significantly and positively associated with high sero-prevalence of IgG antibody to CHIKV and YFV, respectively.

**Conclusion:**

High sero-prevalence of IgG antibody to CHIKV shows the circulation of the virus in the present study area. A low sero-prevalence of IgG antibody to YFV in YF vaccine received individuals is highly concerning from a public health point of view as waning of immune response to YFV infection could result in a periodic outbreaks of YF in endemic areas.Nevertheless, the present study has not investigated for possible cross-reactivity of antibody to CHIKV with other alphaviruses like O’nyong-nyong virus and antibody to YFV with other flaviviruses like Dengue fever virus and this warrants further studies in the present study area.

## Author summary

Mosquito-borne viral diseases including yellow fever and chikungunya are becoming major public health problem in Africa. Community-based sero-epidemiological studies of mosquito-borne viral infections are important to known the occurrence of these diseases and to design appropriate prevention and control strategies. In this study, we assessed the sero-prevalence of IgG antibody against Chikungunya virus and Yellow fever virus in 360 study participants and associated risk factors among the community members of South Omo, Southern Ethiopia. Our study showed a sero-prevalence of IgG antibody; 43.6%, (157 out of 360) to Chikungunya virus, and 49.5% (155 out of 313) against Yellow fever virus in the study participants.Further studies on active case detection of chikungunya and raising awareness, advocating policies to mitigate the risk of arboviral infections have paramount importance in the present study area.

## Introduction

Emerging and re-emerging mosquito-borne viral diseases such as yellow fever (YF), dengue fever (DF) [1], zika, and chikungunya (CHIK), are not only major public health concerns, but also create significant economic problems in many tropical and subtropical countries [2–4]. Most of these arthropod-borne viruses (arboviruses) have a complex dual-host tropism and circulate between mosquitoes, birds and mammals including humans [5, 6]. The symptoms of CHIK and YF and other arboviral diseases are remarkably similar and are often misdiagnosed as malaria or bacterial infections. The increasing outbreaks of these arboviruses need a comprehensive reappraisal of epidemiological studies and establishing appropriate laboratory diagnostic tests that can differentiate between these viruses in endemic regions [2].

In Ethiopia, YF outbreaks have been reported since the 1960s which resulted in many morbidities and mortalities in the southern parts of the country [7, 8]. More recently, in 2012 and 2013, YF outbreak re-occurred in South Omo Zone [9]. With regards to other arboviruses, there is a report of the occurrence of DF in 2013 in the eastern parts of Ethiopia [10–12] and an outbreak of CHIK in 2019 in the northeast and eastern parts of Ethiopia where there is no previous history for the occurrence of CHIK. These evidences suggest that Ethiopia faces a risk of emerging and re-emerging mosquito-borne viral diseases in areas where environmental conditions are suitable for mosquito vectors.

CHIK outbreaks have been reported from many African countries including Uganda [13], Kenya [14–16], Sudan [17] and Tanzania [18]. Despite the proximity of Ethiopia to these countries, and migration of people across the border between Ethiopia and contingent countries as well as the existence of the potential mosquito vectors (*Aedes bromeliae* and *Aedes aegypti*) responsible for transmitting CHIKV in the study area [9], and the repeatedly occurrence of CHIK outbreaks in Kenya [14–16, 19, 20], either health facility or community-based survey of CHIKV infection has not been conducted in the present study area.

There was no routine vaccination for YF in the present study area. However, following the 2012/2013 YF outbreak in the South Omo Zone, an emergency mass-vaccination campaign was undertaken in June 2013 among priority target groups of the Zone to control the outbreak. Among 607,462 people targeted for vaccination, 543,558 (89.5%) individuals were vaccinated [21]. Although the mass-vaccination resulted in the control of outbreak in 2012/13, waning immunity could result in YF resurgence in this endemic area, and hence, it is very important to know the sero-prevalence of IgG antibody against YFV in the present study area.

Therefore, in this study, we assessed community-based sero-prevalence of IgG antibody against CHIKV and the sero-status of IgG antibody against YFV in South Omo, southern part of Ethiopia.

## Methods

### Study area and population

The study was conducted in South Omo Zone, one of the 13 zones in Southern Nations, Nationalities and Peoples' Region (SNNPR) of Ethiopia. This Zone is located approximately 750 km to the south of Addis Ababa and borders Kenya to the South. Detailed information about the South Omo Zone and the demographics of that Region has been described elsewhere [22, 23].

Among the eight districts of the Zone, three districts (Debub Ari, BenaTsemay and Hamer) were selected for the present study based on the ecological suitability and increased likelihood for the occurrence of emerging and re-emerging mosquito-borne viral diseases [9]. More detailed information about the study districts and kebeles (smaller administrative units of a district) has been described elsewhere [9, 23].

### Study design, sample size and sampling techniques

Between February and June 2018, a community-based cross-sectional survey was conducted in selected kebeles of the above mentioned three districts. To the best of our knowledge, there is no information about the level of community-based sero-prevalence of IgG antibody to CHIKV in the present study area or in Ethiopia. Assuming 50% sero-prevalence of IgG antibody to CHIKV in the study area, with 95% confidence in the estimate, 5% margin of error and 90% response rate, 385 study participants were included in the study.

Prior to data collection, the selected kebeles were categorized into 3 clusters based on their geographical proximity, and a list of all the households in these clusters was obtained. The total sample size of 385 was allocated proportionally to the number of households of the clusters. The required number of participants from each cluster was selected using systematic random sampling. The inclusion criteria for participation were being resident in the cluster area, being older than 5 years and volunteer to participate in the study. Pregnant women and anemic individuals were not included in the study.

### Data collection and laboratory investigation

Each study participant (parent in the case of children) was interviewed about their occupation, residency and travel history using a questionnaire. Information about socio-demographic characteristics of the participants was included in the questionnaire. From each study participant, 3 ml venous blood was collected into serum separator vacutainer test tube and serum was separated and stored at -20°C until further screening for IgG antibody against CHIKV and YFV by ELISA. The ELISA was conducted as per the manufacturer’s protocol and samples were analyzed in duplicates and read at an optical density (OD) of 450 nm using a 96-well ELISA plate reader. Positive and negative sera provided by the manufacturer were used as controls for each plate.The result was interpreted as positive or negative on the basis of the manufacturer’s recommended cut-off values using the mean absorbance of the positive and negative controls.

According to the information obtained from health professionals at each of the study sites, during the 2013 YF mass-vaccination, all eligible individuals were first registered in the YF vaccination registry book at the nearest health Institution. Following, YF vaccine was given and YF vaccination card (yellow card) was distributed to those individuals who received the vaccine. However, sometimes some registered individuals could refuse to receive the vaccine, or there was a shortage of vaccine or there was a shortage YF vaccination card. Hence, vaccination status for YF was determined as: 1) vaccinated, for those participants who were registered in the vaccination registry book, reported that they received the vaccine (in case of adults or parents in the case of children), and/or have YF vaccination card, 2) unvaccinated, for those participants who were not registered in the YF vaccination registry book, reported that they did not receive the vaccine and have no YF vaccination card, and 3) unknown status, for those participants who were registered in the YF vaccination registry book, but did not remember whether they received the vaccine or not, or have no YF vaccination card.

### Ethical considerations

The study protocol was approved by the Institutional Review Board (IRB) of the Aklilu Lemma Institute of Pathobiology, Addis Ababa University. The aim of the study was explained to each of the study participants/parents and written consent/assent was obtained from each participant/parents. Blood sample collection was carried out under aseptic conditions by experienced medical laboratory technicians.

### Data analysis

Data were entered into Epi Data Software v.3.1 and analyzed using SPSS Version 25. Frequencies and percentages were used to summarize socio-demographic characteristics and the sero-prevalence of IgG antibody elicited towards CHIKV and YFV were estimated by dividing the number of participants with positive test results by the total number of study participants. Univariable logistic regression analysis was performed to assess the association between the sero-prevalence of IgG antibody and the background characteristics of study participants including age, sex, and history of travelling and other variables. Multivariable logistic regression analysis was performed to assess the effect of each of the independent variables (i.e. gender, age, occupation and others) on the outcome variable after adjusting each independent variable for all other variables. All the independent variables (predictors) included in the final model were reported with their coefficients. Analysis results with a P-value of below 0.05 were considered statistically significant.

## Results

### Background characteristics of study participants

The background characteristics of the study participants are summarized in Table 1. A total of 360 study participants (51.7% males, age range from 6 to 80 years, mean age ± SD = 31.95 ± 14.05 years) participated in this study. The majority of the study participants (48.1%) were in the age group of 21–35 years, 38.6% reported prior vaccination for YF (50.9% among age group 21–35 years old), and 50.3% reported a history of travel to forest areas for different activities.

**Table 1:** Background characteristics of the study participants (N = 360).

Characteristics	Category	No. (%)
Sex	Male	186(51.7)
	Female	174(48.3)
Age group (years)	5–10	17(4.7)
	11–20	62(17.2)
	21–35	173(48.1)
	36–55	86(23.9)
	>55	22(6.1)
Educational status	Not attended formal education	230(63.9)
	Attended formal education	130(36.1)
Occupation	Farmer	169(46.9)
	Pastoralist	152(42.2)
	Others	39(10.8)
District	Debub Ari	190(52.8)
	Bena-Tsemay	35(9.7)
	Hamer	135(37.5)
Vaccination status for YF	Vaccinated	139(38.6)
	Not vaccinated	109(30.3)
	Unknown status	112(31.1)
Travel history to jungle area	Yes	229(63.6)
	No	59(16.4)
	Unknown	72 (19.4)

### Clinical symptoms related to arboviral infections as reported by study participants

Ever experience of clinical symptoms related to arboviral infection as reported by study participants are summarized in Table 2. When the study participants were asked if they experienced any signs and symptoms relevant to arboviral infections, 13.6% reported having had joint pain and muscle aches, while 6.1% reported signs of weakness and fatigue and 2.2% reported non-specific symptoms like fever, headache and body rash. Joint pain and muscle aches were the top symptoms reported by the participants.

**Table 2:** Clinical symptoms reported by the participants (N = 360).

Clinical symptoms	Number (%)
Joint pain and muscle aches	49(13.6)
Loss of appetite	8(2.2)
Nausea and vomiting	13(3.6)
Weakness and fatigue	22(6.1)
Dark urine	17(4.7)
Yellowish skin and eye (jaundice)	8(2.2)
Non-specific symptoms (fever, headache and body rash)	8(2.2)
Did not report symptoms/signs related to arboviral infections	235 (65.3)

### Sero-prevalence of IgG antibody elicited towards CHIKV

**Fig 1:**
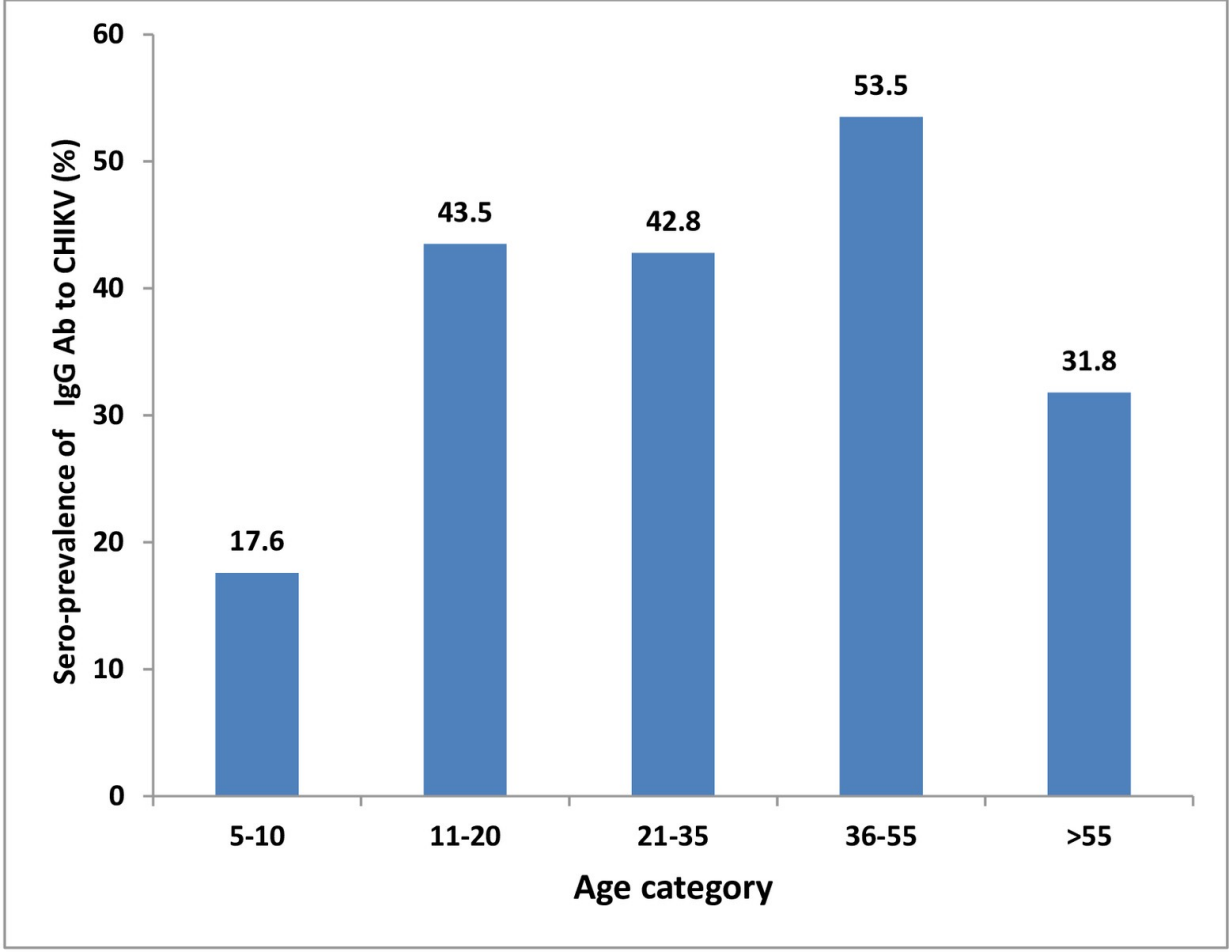
Sero-prevalence of IgG antibody to CHIKV by age category.

The overall sero-prevalence of IgG antibody to CHIKV was 43.6% (39.8% among males and 47.7% among females). The sero-prevalence of IgG antibody to CHIKV was 53.5% (highest) among age group 36–55 and 17.6% (lowest) among the age group of 5–10 years (Fig 1).

In univariable logistic regression analysis a high sero-positivity for IgG antibody to CHIKV was associated with the age group 36–55 of years (COR = 5.37, 95% CI, 1.44, 20.03). However, statistically significant difference was not observed in a multivariable logistic regression analysis (AOR = 0.74, 95% CI, 0.09, 5.75). With respect to the study district, a high sero-prevalence of IgG antibody to CHIKV was observed in participants from Debub Ari (51.6%) compared to other districts (Table 3). In a multivariable logistic regression analysis, an 8-fold higher sero-positivity for IgG antibody to CHIKV was associated with participants from Debub Ari district as compared to those from Hamer district (AOR = 8.47; 95% CI: 1.50, 47.74). Table 3 summarizes the sero-prevalence of IgG antibody to CHIKV and associated factors.

**Table 3:** Sero-positivity for IgG antibody to CHIKV and associated factors in the study participants (N = 360).

Variables	Category	CHIK IgG sero-status		COR (95% CI)	AOR (95% CI)
		Negative (%)	Positive (%)		
Sex	Male	112(60.2)	74(39.8)	0.72(0.48, 1.10)	0.75(0.44, 1.28)
	Female	91(52.3)	83(47.7)	Ref	Ref
Age Group (years)	5–10	14(82.4)	3(17.6)	Ref	Ref
	11–20	35(56.5)	27(43.5)	3.60(0.94, 13.81)	0.28(0.03, 2.52)
	21–35	99(57.2)	74(42.8)	3.49(0.97, 12.58)	0.39(0.05, 3.13)
	36–55	40(46.5)	46(53.5)	5.37(1.44, 20.03)	0.74(0.09, 5.75)
	>55	15(68.2)	7(31.8)	2.18(0.47, 10.12)	0.45(0.04, 4.69)
Educational Status	Not attended FE	136(59.1)	94(40.9)	0.74(0.48, 1.13)	0.74(0.41, 1.35)
	Attended FE	67(51.5)	63(48.5)	Ref	Ref
Occupation	Farmer	85(50.3)	84(49.7)	0.94(0.47, 1.88)	0.71(0.26, 1.94)
	Pastoralist	99(65.1)	53(34.9)	0.51(0.25, 1.04)	0.52(0.16, 1.68)
	Others	19(48.7)	20(51.3)	Ref	Ref
District	Debub Ari*	92(48.4)	98(51.6)	1.81(1.15, 2.84)	8.47(1.50, 47.74)
	BenaTsemay	26(74.3)	9(25.7)	0.59(0.26,1.37)	3.30(0.56, 19.34)
	Hamer	85(63.0)	50(37.0)	Ref	Ref
Travel history to jungle area	Yes	96(53.0)	85(47.0)	0.84(0.43, 1.63)	0.99(0.49, 2.01)
	No	107(59.8)	72(40.2)	Ref	Ref
Joint pain and muscle aches	Yes	25(51.0)	24(49.0)	1.29(0.70, 2.35)	3.16(0.62, 16.15)
	No	178(57.2)	133(42.8)	Ref	Ref
Nausea and vomiting	Yes	9(69.2)	4(30.8)	0.56(0.17, 1.87)	0.62(0.04, 10.10)
	No	194(55.9)	153(44.1)	Ref	Ref
Weakness and fatigue	Yes	11(50.0)	11(50.0)	1.32(0.56, 3.12)	4.00(0.68, 23.66)
	No	192(56.8)	146(43.2)	Ref	Ref
Non-specific symptoms	Yes	5(62.5)	3(37.5)	0.77(0.18, 3.28)	4.98(0.61, 40.91)
	No	198(56.3)	154(43.8)	Ref	Ref

CI (confidence interval), COR (crude odds ratio), AOR (adjusted odds ratio), FE (formal education), Ref (reference category) and

*statistically significant at p<0.05

### Sero-prevalence of IgG antibody elicited towards YFV

**Fig 2:**
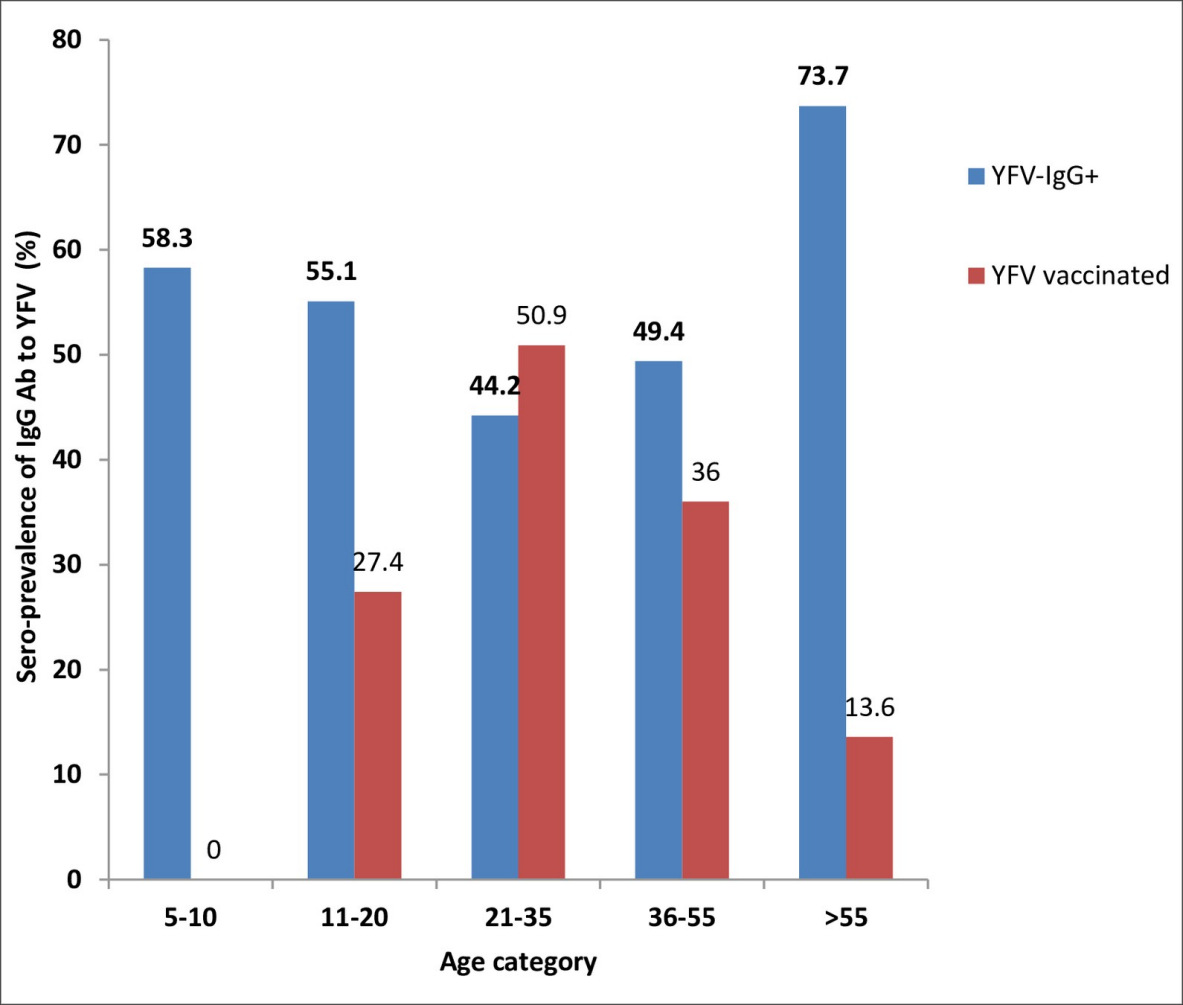
Sero-prevalence of IgG antibody to YFV among YF vaccinated (N = 139) and unvaccinated (N = 109) participants by age category.

Out of 313 analyzed samples, 155 (49.5%) were tested positive for IgG antibody to YFV. The sero-prevalence was 47.8% among males and 51.3% among females. The sero-prevalence was 73.7% (highest) in the age group >55 years old and 44.2% (lowest) in the age group 21–35 years old (Fig 2).

However, age group >55 years old was not associated with a high seropositivity for IgG antibody to YFV in both univariable and multivariable logistic regression analyses (Table 4).The sero-prevalence was 36.3% among vaccinated, 49.0% among unvaccinated and 74.3% among unknown vaccine status. Table 4 shows the sero-prevalence of IgG antibody to YFV and associated factors. In multivariable logistic regression analysis, a 2-fold higher sero-positivity for IgG antibody to YFV was associated with travel history to forest areas compared to individuals who did not (AOR = 2.21; 95% CI: 1.02, 4.81) (Table 4).

**Table 4:** Sero-positivity for IgG antibody to YFV and associated factors in the study participants (N = 313).

Variables	Category	YFV IgG sero-status		COR (95% CI)	AOR (95% CI)
		Negative (%)	Positive (%)		
Sex	Male	83(52.2)	76(47.8)	0.87(0.56, 1.35)	0.82(0.47, 1.44)
	Female	75(48.7)	79(51.3)	Ref	Ref
Age Group	5–10	5(41.7)	7(58.3)	Ref	Ref
	11–20	22(44.9)	27(55.1)	0.88(0.24, 3.15)	0.55(0.06, 4.79)
	21–35	87(55.8)	69(44.2)	0.57(0.17, 1.86)	0.40(0.05,2.96)
	36–55	39(50.6)	38(49.4)	0.70(0.20, 2.39)	0.61(0.09, 4.43)
	>55	5(26.3)	14(73.7)	2.00(0.43, 9.29)	1.46(0.16, 13.66)
Educational Status	Not attended FE	88(45.6)	105(54.4)	1.67(1.05, 2.65)	0.95(0.51, 1.74)
	Attended FE	70(58.3)	50(41.7)	Ref	Ref
Occupation	Farmer	101(59.8)	68(40.2)	0.81(0.38, 1.71)	1.41(0.49, 4.09)
	Pastoralist	39(35.1)	72(64.9)	2.22(1.01, 4.87)	1.56(0.48, 5.08)
	Others	18(54.5)	15(45.5)	Ref	Ref
District	Debub Ari	116(61.1)	74(38.9)	0.24(0.14, 0.42)	0.73(0.14, 3.83)
	BenaTsemay	18(51.4)	17(48.6)	0.35(0.16, 0.80)	0.73(0.14, 3.79)
	Hamer	24(27.3)	64(72.7)	Ref	Ref
YF vaccination status	Vaccinated	86(63.7)	49(36.3)	0.59(0.35, 1.00)	0.74(0.41, 1.35)
	Unknown	19(25.7)	55(74.3)	3.01(1.57, 5.75)	1.46(0.16, 13.66)
	Not vaccinated	53(51.0)	51(49.0)	Ref	Ref
Travel history to jungle area	Yes*	102(56.0)	80(44.0)	2.28(1.08, 4.81)	2.21(1.02, 4.81)
	No	32(74.4)	11(25.6)	Ref	Ref
Joint pain and muscle aches	Yes	7(20.0)	28(80.0)	4.76(2.01, 11.25)	3.69(0.31, 44.52)
	No	151(54.3)	127(45.7)	Ref	Ref
Nausea and vomiting	Yes	3(37.5)	5(62.5)	1.72(0.40, 7.33)	1.08(0.07, 17.02)
	No	155(50.8)	150(49.2)	Ref	Ref
Weakness and fatigue	Yes	3(20.0)	12(80.0)	4.34(1.20, 15.68)	0.82(0.05,13.26)
	No	155(52.0)	143(48.0)	Ref	Ref
Non-specific symptoms	Yes	2(33.3)	4(66.7)	2.07(0.37, 11.45)	1.41(0.14, 13.85)
	No	156(50.8)	151(49.2)	Ref	Ref

CI (confidence interval), COR (crude odds ratio), AOR (adjusted odds ratio), FE (formal education), Ref (reference category) and

*(significant at p<0.05)

### Sero-prevalence of IgG antibody to CHIKV among YFV IgG antibody positive and negative study participants

**Fig 3:**
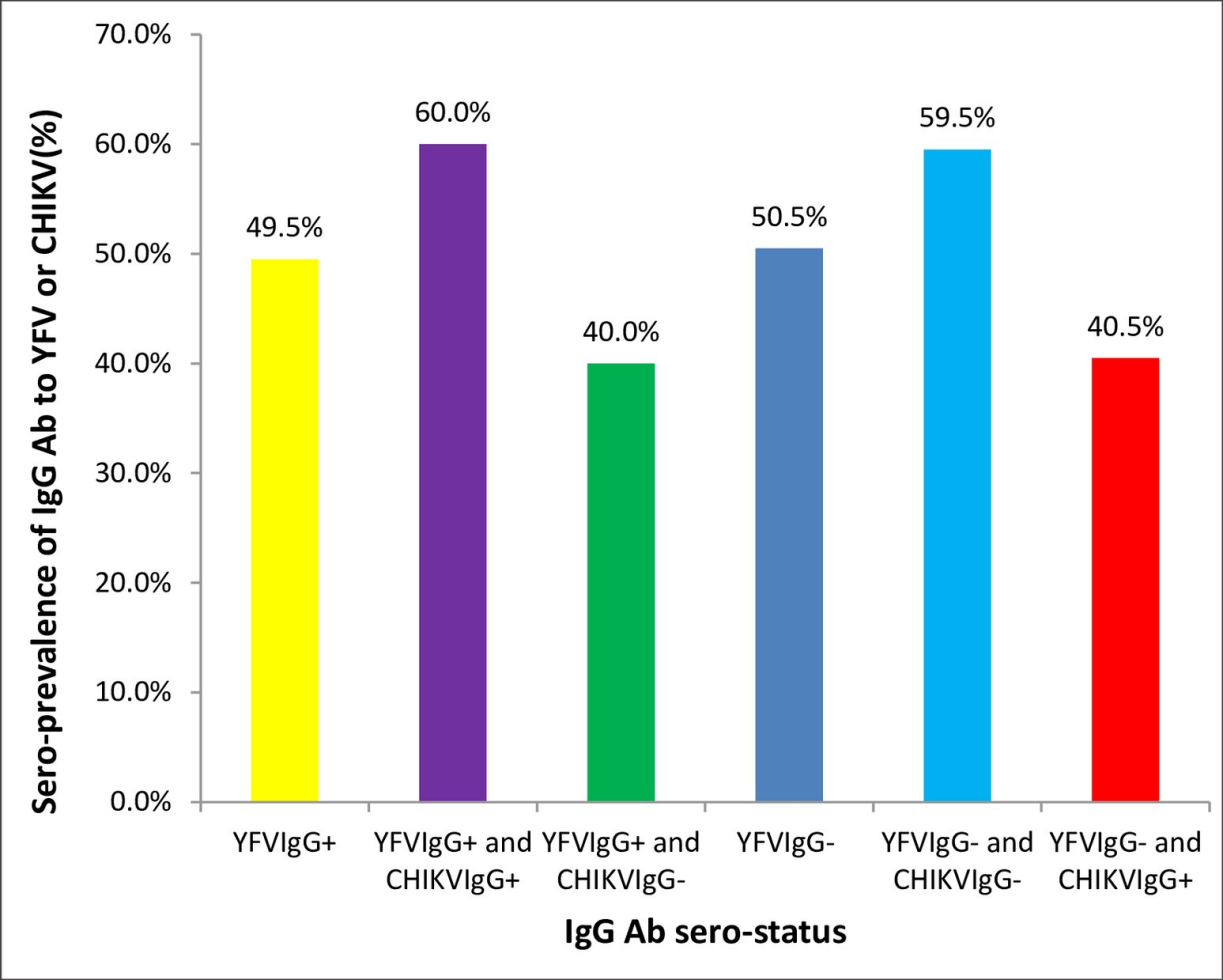
Sero-prevalence of IgG antibody against CHIKV among YFV IgG antibody sero-positive and sero-negative participants.

Out of the 155 YFV IgG antibody positive samples, 93(60.0%) were positive for IgG antibody to CHIKV while, out of 158 samples which were negative for IgG antibody to YFV, 64(40.5%) were positive for IgG antibody to CHIKV. Fig 3 shows the sero-prevalence of IgG antibody to CHIKV among YFV IgG antibody positive and negative study participants.

### Correlation of the presence of CHIKV IgG antibody with YFV IgG antibody in the study participants

**Fig 4:**
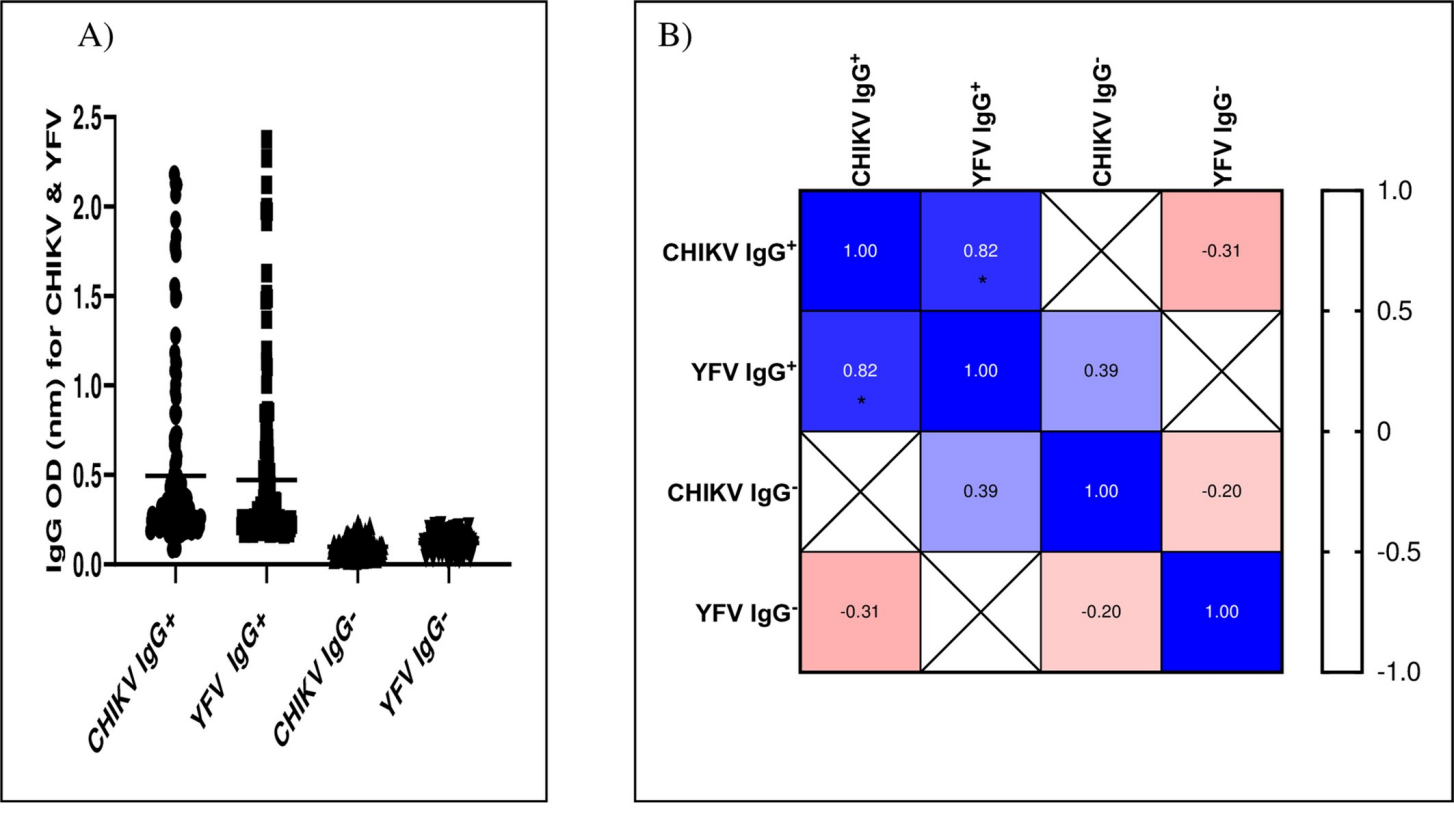
**A & B**. Correlation of IgG antibody to CHIKV with IgG antibody to YFV among the study participants.

The correlation analysis showed a significant positive correlation between anti-CHIKV IgG^+^ and anti-YFV IgG^+^ antibodies (rs = 0.82; P<0.01) (Fig 4A & 4B).

**Fig 5:**
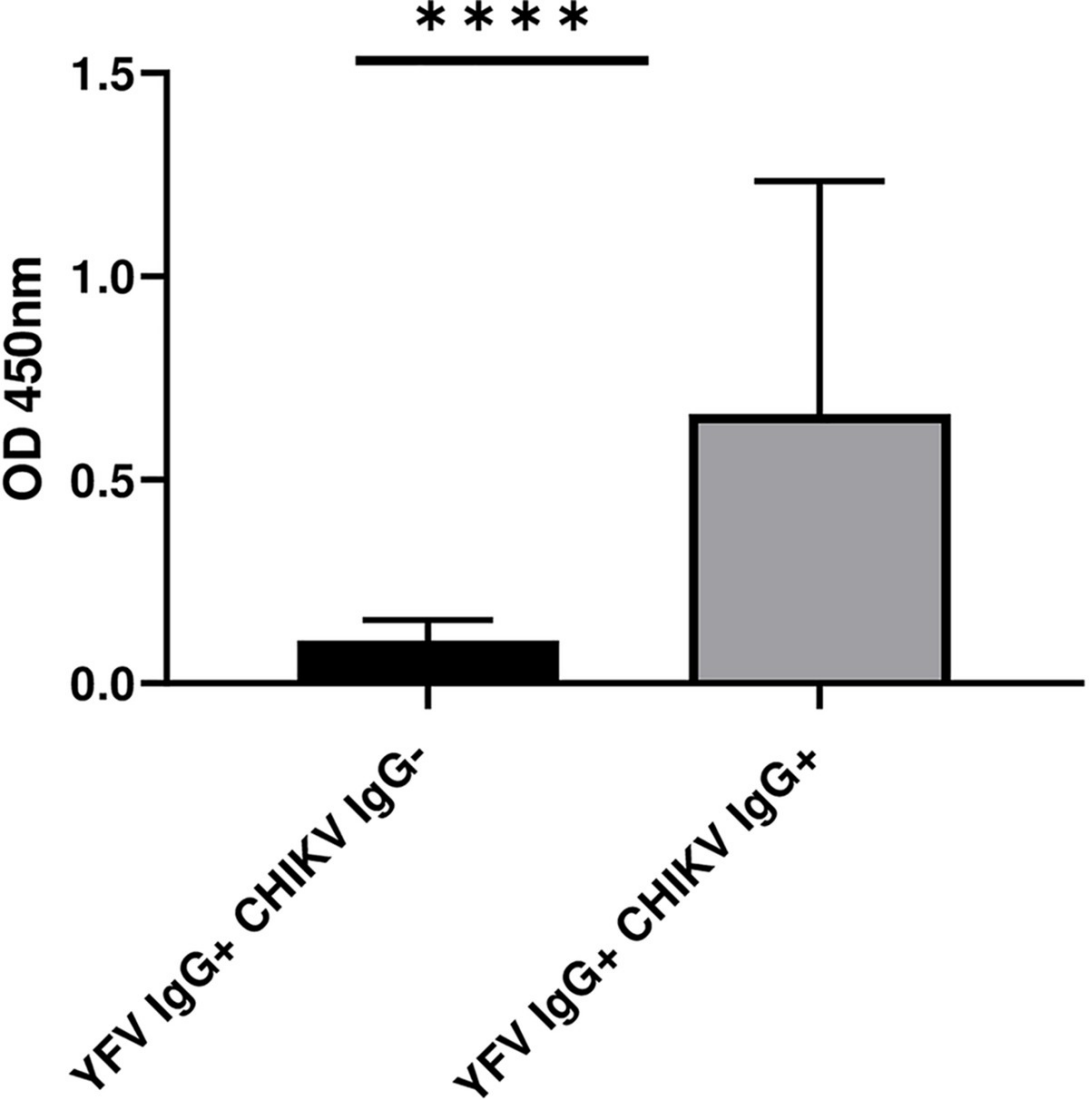
Comparison of the OD values of YFV IgG^+^ samples stratified by the presence or absence of CHIKV IgG antibody (CHIKV IgG^+^ or IgG^-^).

In addition, the YFV IgG^+^ titer was higher in samples that were also positive for CHIKV IgG antibody as compared to negative samples (Fig 5).

## Discussion

The aim of this study was to investigate the community-based sero-prevalence of CHIKV and YFV in the South Omo Zone of Ethiopia, an endemic area for YFV.The current findings show high sero-prevalence of CHIKV infection (43.6%) in the study participants as detected by IgG capture ELISA suggesting past and/or current circulation of the virus in the study area. The proportion of the population exposed to CHIKV infection in the current study is considerably larger as compared to the IgG antibody sero-prevalence studies reported from rural coastal areas of Kenya [15], Tanzania [24], Singapore [25], Brazil [26], nomadic pastoralist of Senegal [27] and in other African countries [28]. The possible justification for such a high sero-prevalence of CHIKV infection in the present study area might be due to the presence of conducive local environmental conditions that favor vector competence in transmitting the disease [9, 29]. Thus, the presence of favorable mosquito breeding sites and frequent contact between humans and wildlife in the human-animal transmission interface of rural settings could increase the chance of viral spillover to humans [29,30]. In addition, the present study area has been known to be endemic for YF since the 1960s [7, 8], and hence, mosquitoes and non-human primates which maintain the natural transmission cycle of YFV could also serve as reservoirs for CHIKV and for many other arboviruses. The proximity of the present study area to Kenya, a country reported the occurrence of CHIKV infection [14–16, 20] could have resulted in an increased sero-prevalence of CHIK in the present study area since migration and travel of people across the border inferring the likelihood of transmission of this virus.

A relatively higher sero-prevalence (53.5%) of IgG antibody against CHIKV was observed in the older age group (36–55 years) of the study participants which implies stable endemicity is accompanied by increasing seroprevalence as age increased. Several previous studies also showed a higher percentage of sero-prevalence of IgG antibody against many arboviruses in older individuals [20, 24, 26]. In the present study, a higher sero-positivity for IgG antibody to CHIKV was also observed in participants from Debub Ari district as compared to participants from other districts. This across district variability of the risk of infection in the current study highlights the importance of designing interventions for different localities based on their level of risks. Our result is in agreement with a previous study which showed that Debub Ari district was the highly affected during the YF outbreak in 2013 [9]. This might be attributed to geographic/ecological factors and/or human activities. For various activities, many of the inhabitants of Debub Ari district are migrating to the forest areas such as Mago National Park and Maki areas where there are a large number of non-human primates and also favorable environment for the breeding of mosquitoes responsible for sylvatic arboviruses transmission [30]. This movement of the inhabitants of Debub Ari district to forest areas and share the same environment with non-human primates could maintain and promote the transmission of certain arboviruses.

The current study was conducted five years after YF mass vaccination campaign for targeted age groups [21] following the 2012/013 YF outbreak in the study area. Out of 313 tested samples for IgG antibody against YFV infection/vaccine, 155 (49.5%) were tested positive for IgG antibody to YFV infection/vaccine. The observed sero-prevalence of IgG antibody against YFV in the current study area could be either due to the recent vaccination and/or due to natural infection, as the area is known to be an endemic site for YF [7–9]. A study by Staples et al. [31] showed a seroprevalence of 13.3% IgG antibody (naturally–acquired antibodies) against YFV in individuals who did not receive YF vaccine in YF endemic area of Central African Republic.

It has been reported that more than 80% of the population in the present study area was vaccinated against YFV in 2013 [21]. However, the observed overall sero-prevalence (49.5%) of IgG antibody against YFV was low compared to the results of other sero-prevalence studies conducted in YF vaccinated populations in Sudan [32], Brazil [33] and other country [34]. On the other hand, the sero-prevalence against YFV in our study was still higher than the national pooled sero-prevalence of Ethiopia [35] and Western Kenya [19]. These geographic based differences in the sero-prevalence of IgG antibody in populations which were vaccinated for YF in African countries call for further studies.

In the present study, the sero-prevalence of IgG antibody was found to be higher among the study participants who did not receive YF vaccine (49.0%) or who did not know their vaccine status (74.3%) compared to vaccinated individuals (36.3%). Interestingly, these results indicate that in YF endemic areas of Africa most of the inhabitants are likely protected against YFV infection progression to severe cases due to naturally-acquired antibodies against YFV [31]. It has been suggested that a single dose of YF vaccine can confer a protective immunity to YFV for lifelong [1], On the other hand, the sero-negativity of IgG antibody in vaccinated participants in the current study area is highly concerning from a public health point of view as waning of antibody response to YFV infection could result in a periodic outbreaks of YF in endemic countries [36–38].

Previous studies in rural Kenya [20], Senegal [27], Tanzania [39], Bangladesh [40] and Singapore [25] reported a higher percentage of sero-prevalence of IgG antibody against many arboviruses including YFV in older individuals. Previous study in Ethiopia also showed a higher sero-prevalence of IgG antibody to YFV in individuals in the age category of 40–64 years [35]. A similar trend was also observed in the current study. Most likely this can be attributed to the fact that the risk of viral exposure increases with the age of each individual [40]. Our result also showed that the sero-prevalence of IgG antibody against YFV was higher in the study participants who reported regular travel to sylvatic areas for different activities. A study by Staples *et al*. [31] revealed a high sero-positivity for IgG antibody to YFV in residents bordering forest areas in the Central African Republic. This result might be explained by the fact that the virus circulates between mosquitoes and non-human primates in the sylvatic area and accidentally transmits to humans who visits these areas [30, 41].

A previous study showed a low level/absence of cross-reactivity between antibodies to alphaviruses including CHIKV and antibodies to flaviviruses-infected patients [42]. In the present study, majority of the samples (60.0%) which were positive for IgG antibody to YFV were also positive for IgG antibody to CHIKV and a positive correlation was also observed between the presence of anti-CHIKV IgG^+^ and anti-YFV IgG^+^ antibody. In contrary, the higher YFV IgG^+^ titer was observed among CHIKV IgG positive samples as compared to the negative samples; this might indicate co-circulation of the viruses at the same time or circulation of the viruses at different times in the study area rather than the effect of cross-reaction. However, the findings warrant further studies to come-up with a better understanding of the correlation between CHIKV and YFV IgG^+^ antibodies.

### Limitations of the study

As to the best of our knowledge, this is the first community-based study conducted in Ethiopia to assess the sero-prevalence of CHIKV infection in YF endemic area. It is also the first community based seroprevalence study of IgG antibody to YFV in a mass YF vaccinated population in the country. However, the findings of our study have limitations which warrant further studies. First, although a high sero-prevalence of IgG antibody against CHIKV was observed, a possible cross-reactivity of CHIKV with other related alphaviruses including O’nyong-nyong virus (ONNV) and Semliki Forest virus (SFV) [42–44] has not been addressed. Since ONNV outbreaks have occurred in East Africa [45, 46], there is a possibility that many of the CHIKV-seropositive persons had been infected by ONNV. Second, the observed sero-prevalence of IgG antibody against YFV in the current study area could be either due to the recent vaccination and/or due to natural infection. Third, a possible cross-reactivity of YFV with other flaviviruses including Dengue fever virus, Zika virus and West nile virus which could reduce the specificity of the detected sero-positivity of IgG antibody to YFV in endemic areas [47, 48] has not been addressed using a confirmatory test such as a Plaque Reduction Neutralization Test (PRNT) due to the limited availability of resources. The present study area has been well known as YF endemic area since the 1960s outbreaks. However, to our knowledge cases of other flaviviruses have not been reported from the present study areas though there is a report on the occurrence of DF in 2013 (for the first time in Ethiopia) in the eastern parts of the country [9]. During the 2012/13 YF outbreak in the present study area, Lilay et al [9], have collected serum samples from patients suspected of YF and other arboviruses, tested using ELISA as well as confirmatory tests (Plaque Reduction and Neutralization and Real time PCR). They reported that none of the samples was positive for other arboviruses (DF, West nile virus, Zika virus, Rift Valley fever, Crimean-Congo hemorrhagic fever and (CHIK) other than YFV infection. Fourth, the current study has not attempted to detect acute CHIKV and YFV cases among study participants who complained various symptoms using IgM antibody ELISA based assays and this warrants further study. Nevertheless, we believe that our study would provide a baseline data on the epidemiology of CHIK and on the status of IgG antibody response to YFV in a mass YF vaccine received population where these data are not available, but crucial to predict the probability of an outbreak and make necessary preparations.

In conclusion, a community-based sero-prevalence study of arboviral infection during non-outbreak periods in risk areas is crucial to provide information on the status of transmission, to predict the probability of an outbreak and also to make necessary preparations to control and prevent future outbreaks. Although the concern of cross-reactivity of antibody to CHIKV with other alphaviruses like ONNV and SFV has not been addressed, remarkably a high sero-prevalence of IgG antibody against CHIKV in the current study is suggestive of endemic circulation of the virus in the study area. Future studies in this area of Ethiopia should be considered to detect acute CHIKV cases among suspected patients using a combination of qRT-PCR and IgM based assays. Although more than 38% of the participants were vaccinated against YF, low sero-prevalence of IgG antibody against YFV was detected. This implies a potential risk indicator for the reappearance of YF outbreak in the study area and it warrants making necessary preparations to control and prevent future outbreaks. Nevertheless, the present study has not addressed possible cross-reactivity of CHIKV with other alphaviruses like ONNV and YFV with other flaviviruses like Dengue fever virus and this also warrants further studies in the present study area.

## Supporting information

S1 Checklist STROBE checklist of items that should be included in reports of *cross-sectional*. (DOC)

S1 Questionnaire Questionnaire used for the study Community-based sero-prevalence of chikungunya and yellow fever in the South Omo Valley of Southern Ethiopia. (DOCX)
