# Supplementary material for: Community-based sero-prevalence of chikungunya and yellow fever in the South Omo Valley of Southern Ethiopia
Source: PLoS Negl Trop Dis. 2020 Sep 3;14(9):e0008549. doi: 10.1371/journal.pntd.0008549 (PMC7470273; doi:10.1371/journal.pntd.0008549)
Supplement: S1 Checklist — (DOC) [file pntd.0008549.s001.doc]

STROBE Statement—Checklist of items that should be included in reports of ***cross-sectional studies***

|  | Item No | Recommendation |
| --- | --- | --- |
| **Title and abstract** | 1 | (*a*) Indicate the study’s design with a commonly used term in the title or the abstract:  Indicated in the abstract, paragraph 2 |
|  |  | (*b*) Provide in the abstract an informative and balanced summary of what was done and what was found:  Indicated in the abstract, paragraph 2 and 3 |
| Introduction | | |
| Background/rationale | 2 | Explain the scientific background and rationale for the investigation being reported:  Indicated in the introduction, paragraph 1-4 |
| Objectives | 3 | State specific objectives, including any pre-specified hypotheses:  Indicated in the introduction section , paragraph 4 |
| Methods | | |
| Study design | 4 | Present key elements of study design early in the paper:  Indicated in the materials and methods section, sub-heading ‘Study design, sample size and sampling techniques’, paragraph 1 |
| Setting | 5 | Describe the setting, locations, and relevant dates, including periods of recruitment, exposure, follow-up, and data collection:  Indicated in the methods section, sub-heading ‘Study area and population’, paragraph 1-2 and sub-heading ‘Study design, sample size and sampling techniques’, paragraph 1 |
| Participants | 6 | (*a*) Give the eligibility criteria, and the sources and methods of selection of participants:  Indicated in the methods section, sub-heading ‘Study design, sample size and sampling techniques’, paragraph 2 |
| Variables | 7 | Clearly define all outcomes, exposures, predictors, potential confounders, and effect modifiers. Give diagnostic criteria, if applicable:  Indicated in the methods section, under the sub-heading of ‘Data collection and laboratory investigation’, paragraph 1 |
| Data sources/ measurement | 8* | For each variable of interest, give sources of data and details of methods of assessment (measurement). Describe comparability of assessment methods if there is more than one group:  Indicated in the methods section, sub-heading ‘Data collection and laboratory investigation’, paragraph 1 |
| Bias | 9 | Describe any efforts to address potential sources of bias:  Indicated in the methods section, under the sub-heading of ‘Study design, sample size and sampling techniques’, paragraph 2 |
| Study size | 10 | Explain how the study size was arrived at:  Indicated in the methods section, sub-heading ‘Study design, sample size and sampling techniques’, paragraph 1 |
| Quantitative variables | 11 | Explain how quantitative variables were handled in the analyses. If applicable, describe which groupings were chosen and why: Indicated in the methods section, under the sub-heading of ‘Data analysis”, paragraph 1 |
| Statistical methods | 12 | (*a*) Describe all statistical methods, including those used to control for confounding:  Indicated in the methods section, sub-heading ‘Data analysis”, paragraph 1 |
|  |  | (*b*) Describe any methods used to examine subgroups and interactions |
|  |  | (*c*) Explain how missing data were addressed |
|  |  | (*d*) If applicable, describe analytical methods taking account of sampling strategy |
|  |  | (*e*) Describe any sensitivity analyses |
| Results | | |
| Participants | 13* | (a) Report numbers of individuals at each stage of study— e.g. numbers potentially eligible, examined for eligibility, confirmed eligible, included in the study, completing follow-up, and analyzed: Indicated in the result section, sub-heading ‘Background characteristics of study participants’, paragraph 1 |
|  |  | (b) Give reasons for non-participation at each stage |
|  |  | (c) Consider use of a flow diagram |
| Descriptive data | 14* | (a) Give characteristics of study participants (e.g. demographic, clinical, social) and information on exposures and potential confounders:  Indicated in the result section, Table 1 |
|  |  | (b) Indicate number of participants with missing data for each variable of interest |
| Outcome data | 15* | Report numbers of outcome events or summary measures: Reported in the result section, Table 2-4 and supporting information (figures), Fig. 1-3 |
| Main results | 16 | (*a*) Give unadjusted estimates and, if applicable, confounder-adjusted estimates and their precision (eg, 95% confidence interval). Make clear which confounders were adjusted for and why they were included:  Indicated in the result section, Table 3 and 4 |
|  |  | (*b*) Report category boundaries when continuous variables were categorized:  Indicated the result section, Table 1, 3 and 4 and supporting information (figures), Fig. 1-3 |
|  |  | (*c*) If relevant, consider translating estimates of relative risk into absolute risk for a meaningful time period |
| Other analyses | 17 | Report other analyses done—eg analyses of subgroups and interactions, and sensitivity analyses |
| Discussion | | |
| Key results | 18 | Summarise key results with reference to study objectives:  Indicated in the discussion part, paragraph 1-5 |
| Limitations | 19 | Discuss limitations of the study, taking into account sources of potential bias or imprecision. Discuss both direction and magnitude of any potential bias:  Indicated after the discussion section, sub-heading ‘Limitations of the study’, paragraph 1 |
| Interpretation | 20 | Give a cautious overall interpretation of results considering objectives, limitations, multiplicity of analyses, results from similar studies, and other relevant evidence:  Indicated in the discussion part, paragraph 1-5 |
| Generalisability | 21 | Discuss the generalisability (external validity) of the study results:  Indicated in the discussion part, paragraph 1-5 |
| Other information | | |
| Funding | 22 | Give the source of funding and the role of the funders for the present study and, if applicable, for the original study on which the present article is based:  The study was financially supported by the Office of Vice President for Research and Technology Transfer, Addis Ababa University, Addis Ababa, Ethiopia (Ref No. RD/PY 662/2016) and laboratory kits (ELISA) was provided by University of California, Barkley. |

*Give information separately for exposed and unexposed groups.

**Note:** An Explanation and Elaboration article discusses each checklist item and gives methodological background and published examples of transparent reporting. The STROBE checklist is best used in conjunction with this article (freely available on the Web sites of PLoS Medicine at http://www.plosmedicine.org/, Annals of Internal Medicine at http://www.annals.org/, and Epidemiology at http://www.epidem.com/). Information on the STROBE Initiative is available at [www.strobe-statement.org](http://www.strobe-statement.org/).
