# Supplementary material for: Community-based sero-prevalence of chikungunya and yellow fever in the South Omo Valley of Southern Ethiopia
Source: PLoS Negl Trop Dis. 2020 Sep 3;14(9):e0008549. doi: 10.1371/journal.pntd.0008549 (PMC7470273; doi:10.1371/journal.pntd.0008549)
Supplement: S1 Questionnaire — (DOCX) [file pntd.0008549.s002.docx]

**Questionnaire used for the study Community-based sero-prevalence of chikungunya and yellow fever in the South Omo Valley of Southern Ethiopia**

**Name of Participant:** _______________________________**No.________________________**

**Woreda________ Kebele ____________Village___________ Date _____/_____/_________**

***Please kindly provide information for the following general questions***

1. Sex: 1. Male 2. Female
2. Age: _________________ years
3. Ethnicity: 1. Ari 2. Bena 3. Tsemay 4. Hamer 5. Others (specify) ___________
4. Religion: 1. Orthodox 2. Muslim 3. Protestant 4. Catholic 5. Others(specify) ______
5. Educational Status: 1. Illiterate 2. Read only 3. Read and write 4. Secondary & above 5. Other ___________
6. Occupation: 1. Farmer 2. Nomadic pastoralist 3. Agro pastoralist 4. Others (specify)___
7. Duration of stay in this kebele: _____________
8. History of residence in other areas 1.Yes 2. No
9. If Q8 yes, where and when: Where ______________When_____________months/years
10. Do you have any travel history to other area: 1. Yes 2. No
11. If yes where and when: Where __________________; When ___________________
12. History of working/traveling to areas like Mago park area, Shala, Luka areas: 1. Yes 2. No
13. If Q12 yes, when and for what purpose: When _________; Purpose ____________
14. Do you know this mosquito (Aedes mosquitoes’ picture): 1. Yes 2. No
15. History of biting by this mosquito (Aedes mosquitoes’ picture): 1. Yes 2. No
16. If Q15 yes, where and when: 1. Night at home; 2. Day time around home; 3. Day time in Forest area /Mago park area; 4. Other specify _____________
17. If Q15 yes, do you think biting by this mosquito causes a disease: 1. Yes 2. No 3. I don’t know
18. If Q17 yes, symptoms of the disease: 1. Severe/high headache 2. Vomiting 3. Muscle pain 4. Yellowish eye 5. Generalized bleeding 6. Encephalitis 7. Dark urine 8. Other _____
19. If Q17 yes, The symptoms persist for how many days: _________________
20. If Q17 yes, does it have a treatment: 1. Yes 2. No 3. I don’t know
21. If Q20 yes, its treatment: 1. Modern 2. Traditional medicinal plant 3. Other ________
22. Any history of current illness: 1. Yes 2. No
23. If Q22 yes, symptoms of the current illness: 1. Severe/high headache 2. Vomiting 3. Muscle pain 4. Yellowish eye 5. Generalized bleeding 6. Encephalitis 7. Dark urine 8. Other ____
24. If Q22 yes duration/onset of illness: __________________ days
25. Body temperature : _________________________
26. Treatment sought: ___________________________
27. Any history of chronic diseases: 1. Yes 2. No
28. If Q27 yes, mention the disease: __________________
29. History of vaccination for yellow fever (check list/card): 1. Yes 2. No
